# Lignin-Based N-Carbon Dots Anchoring NiCo_2_S_4_/Graphene Hydrogel Exhibits Excellent Performance as Anodes for Hybrid Supercapacitor

**DOI:** 10.3390/polym16212959

**Published:** 2024-10-22

**Authors:** Linlin Cui, Hanping Xu, Long Zhang, Xiaojuan Jin

**Affiliations:** 1 Goertek College of Science and Technology Industry, Weifang University, Weifang 261061, China; 2 Beijing Key Laboratory of Lignocellulosic Chemistry, MOE Key Laboratory of Wooden Material Science and Application, MOE Engineering Research Center of Forestry Biomass Materials and Bioenergy, Beijing Forestry University, Beijing 100083, China

**Keywords:** NiCo_2_S_4_, lignin, lignin-based N-carbon dots, supercapacitors

## Abstract

A NiCo_2_S_4_/N-CDs/RGO ternary composite hydrogel was prepared via a one-step hydrothermal method, utilizing lignin-based nitrogen-doped carbon dots as a bridge connecting NiCo_2_S_4_ and graphene. The specific capacitance of NiCo_2_S_4_/N-CDs/RGO significantly outperforms that of the GH and NiCo_2_S_4_/RGO electrodes, achieving 1050 F g^−1^. The 3D mesh porous hydrogel structure mitigates NiCo_2_S_4_ nanoparticle aggregation, providing a larger specific surface area for enhanced charge storage. The abundant functional groups of N-CDs interact with Ni (II) and Co (III) cations, favoring NiCo_2_S_4_ particle synthesis. Additionally, an assembled solid-state asymmetric supercapacitor employing NiCo_2_S_4_/N-CDs/RGO as the positive electrode exhibited excellent energy density (68.4 Wh kg^−1^) and cycle stability (82% capacitance retention after 10,000 constant current charge–discharge cycles).

## 1. Introduction

As energy conversion/storage devices, electrochemical energy storage devices can effectively address the temporal and spatial limitations of clean energy such as solar, wind, and tidal power, promoting the development of new energy vehicles, aerospace, and intelligent electronic devices [1,2,3,4,5]. Therefore, developing high-performance electrochemical energy storage devices is a current research focus. Among many energy storage devices, supercapacitors have attracted considerable attention due to long service life, wide operating temperature range, fast response speed, and high power density [6,7,8,9]. As core components of supercapacitors, designing and constructing high-performance electrodes directly determines the energy storage performance of the entire device [10,11,12].

The electrochemical properties of electrode materials are closely related to the structure and properties of electroactive materials [13]. Transition metal sulfides are widely used as supercapacitor electrode materials due to their diverse nanocrystalline morphology and high theoretical specific capacitance [14,15,16]. Among them, NiCo_2_S_4_ is a representative material. Due to the synergistic action of nickel and cobalt metal ions, NiCo_2_S_4_ exhibits more abundant redox reactions than corresponding binary metal sulfides [17,18,19]. Moreover, compared to NiCo_2_O_4_, NiCo_2_S_4_ has smaller optical band gap energy and higher conductivity [20,21,22]. However, NiCo_2_S_4_ has certain drawbacks, such as a structure that easily collapses and poor cycling stability [23,24]. To effectively solve the issue of structural collapse, one approach involves constructing nanocomposite materials. Carbon materials have the characteristics of good stability and a large specific surface area. Studies show that the in situ growth of NiCo_2_S_4_ nanoparticles on a highly conductive carbon matrix effectively overcomes this shortcoming [20].

Among many carbon materials, graphene has attracted considerable attention. Graphene, a two-dimensional carbon material with a thickness of one atom, is an ideal scaffold for growing functional nanomaterials due to its large specific surface area, good electrical conductivity, and high mechanical flexibility [25,26]. Notably, graphene nanosheets tend to agglomerate under van der Waals forces, resulting in a serious reduction in actual specific surface area and ion transport rate [27]. Three-dimensional (3D) graphene structures can effectively prevent nanosheet agglomeration and increase active sites, offering advantages over two-dimensional graphene in various applications [28,29]. Li et al. [30] successfully synthesized NiCo_2_S_4_ nanoparticles/graphene aerogel (NiCo_2_S_4_/GA), and compared with pure NiCo_2_S_4_ and graphene aerogel, the specific capacitance of NiCo_2_S_4_/GA aerogel improved to 704.34 F g^−1^ at 1 A g^−1^, remaining at 80.3% of the initial specific capacitance after 1500 cycles. Simply mixing NiCo_2_S_4_ with graphene sheets results in poor interface performance due to the weak bonding strength between the two. Therefore, increasing interface contact between NiCo_2_S_4_ nanoparticles and graphene is necessary.

Carbon dots (CDs), new nanomaterials with carbon cores and rich edge functional groups, can combine well with inorganic and organic materials, enhancing the bond strength between different materials [31,32,33,34]. Compared to multi-dimensional carbon materials, zero-dimensional CDs are more flexible due to their small size and easy functionalization [35,36]. More importantly, negatively charged CDs attract metal ions, controlling metal sulfide crystal growth. Currently, CD synthesis mainly uses materials such as small molecular compounds, carbon fibers, graphene, and biomass as initial carbon sources [37]. Among them, preparing carbon dots using resource-rich natural biomass as a precursor has attracted attention [38]. On one hand, biomass carbon dots can reduce costs during preparation; on the other hand, they enable the green recycling of biomass resources, especially waste. Well known as nature’s most abundant aromatic polymer, lignin has high carbon content and unique natural aromatic structure [39,40]. Due to its cheap availability, lignin has become an important precursor for various carbon materials [41,42]. Therefore, preparing carbon dots based on abundant, renewable lignin has good development prospects, aligning with sustainable social development concepts. Furthermore, introducing heteroatoms like N or O on CDs’ surfaces can induce redox reactions, adding pseudocapacitance contributions.

**Figure 1:**
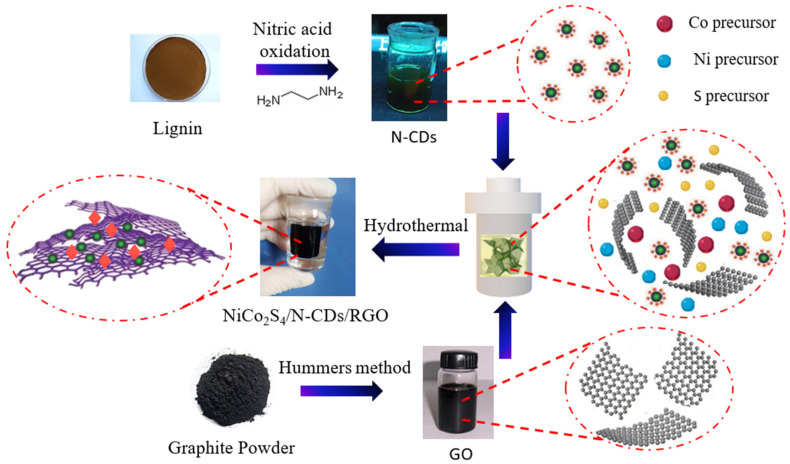
Schematic diagram for the preparation of NiCo_2_S_4_/N-CDs/RGO ternary hydrogel.

In this study, we introduced lignin-based nitrogen-doped carbon dots (N-CDs) to regulate the in situ growth of NiCo_2_S_4_ nanoparticles on graphene nanosheets, and prepared the NiCo_2_S_4_/N-CDs/RGO ternary hydrogel via a one-step hydrothermal method. The experimental preparation process is shown in Figure 1. Nitrogen-doped carbon dots are first anchored in graphene sheets by electrostatic action, while the negatively charged CDs attract metal ions, which in turn promotes the growth of bimetallic sulfide crystals. The reduced graphene oxide (RGO) nanosheets provide a conductive substrate for the carbon dots as well as NiCo_2_S_4_ nanoparticles. Compared to NiCo_2_S_4_/RGO, under the same synthesis conditions, but without N-CDs, NiCo_2_S_4_/N-CDs/RGO exhibits superior electrochemical performance. It is shown that N-CDs not only enhance the bonding strength of NiCo_2_S_4_ and graphene, but also contribute to improving the specific capacitance of the composite due to their electrical conductivity.

## 2. Experimental

### 2.1. Preparation of Graphene Oxide

Graphene oxide was prepared as follows by the classical modified Hummers method [43]: First, 1.5 g of NaNO_3_ was dissolved in 70 mL of H_2_SO_4_, and 3.0 g of graphite powder was added while stirring in an ice-water bath. KMnO_4_ and K_2_FeO_4_ were added to the mixed sulfuric acid solution within a specified time and stirring was continued for 90 min. Under the action of KMnO_4_ and concentrated H_2_SO_4_, graphite intercalation compounds were formed. The mixture was moved to 35 °C water for 3 h, during which the graphite was fully oxidized by concentrated sulfuric acid and KMnO_4_. After the water bath, 150 mL of deionized water was slowly added dropwise along the wall. Concentrated sulfuric acid releases a lot of heat when it meets water, controlling the dripping rate and keeping the temperature below 50 °C, and the reaction system continued to react in a water bath at a temperature of 90 °C for 30 min. In the high-temperature reaction stage, the graphite oxide was exfoliated. The resulting tan suspension was further diluted with 500 mL of warm distilled water, and 15 mL of 30% H_2_O_2_ was added to remove residual KMnO_4_ until a bright yellow suspension was obtained. The obtained solution was washed with a 1:10 hydrochloric acid solution and distilled water in turn, and purified by dialysis and centrifugation until the solution was neutral, and then configured into a 2 mg mL^−1^ graphene oxide solution for later use.

### 2.2. Preparation of Lignin-Based Nitrogen-Doped Carbon Dots

Lignin-based nitrogen-doped carbon dots were prepared by a combination of nitric acid pre-oxidation and hydrothermal carbonization. Using corncob lignin as raw material, 4.0 g of lignin was first added to 20 mL of deionized water and ultrasonically dissolved. Then, 8 mL of 68% nitric acid was added for the oxidative depolymerization of the lignin. The modified lignin was mixed with 6.0 g of ethylenediamine and hydrothermally reacted at 180 °C for 12 h. After the reaction, the upper layer solution was purified by dialysis for 4 days, yielding the nitrogen-doped carbon dots.

### 2.3. Preparation of NiCo_2_S_4_/N-CDs/RGO, N-CDs/RGO and NiCo_2_S_4_/RGO Hydrogel

First, the prepared GO solution and N-CDs were mixed uniformly at a mass ratio of 3:1, along with 365.5 mg NiCl_2_·6H_2_O, 713.7 mg CoCl_2_·6H_2_O, and 913.2 mg CS(NH_2_)_2_. Then, 360 mg CH_4_N_2_O were successively added to the mixed solution. After thorough stirring, the solution was transferred to an autoclave, and hydrothermally heated at 180 °C for 16 h. After cooling to room temperature, the resulting composite hydrogel was washed and dried with ethanol to prepare a NiCo_2_S_4_/N-CDs/RGO hydrogel composite with uniform texture. For comparison, N-CDs/RGO and NiCo_2_S_4_/RGO composites were also prepared using the same experimental method.

### 2.4. Assembly of NiCo_2_S_4_/N-CDs/RGO//GH Asymmetric Supercapacitor

First, 3.0 g PVA was added to 30 mL KOH (3.0 M) solution and stirred at 85 °C until the PVA particles melted and a transparent PVA/KOH gel electrolyte formed. An asymmetric supercapacitor was assembled with NiCo_2_S_4_/N-CDs/RGO and graphene hydrogel (GH) as positive and negative electrodes, respectively. The PVA/KOH gel electrolyte was evenly coated between the two hydrogel electrodes, which were then stacked in parallel and naturally dried until solidifying. Pressing the two electrodes together at ~1.0 MPa yielded NiCo_2_S_4_/N-CDs/RGO//GH capacitors.

### 2.5. Materials Characterizations

The morphology of the samples was characterized using a Zeiss field emission scanning electron microscope (Zeiss Gemini 300, Oberkochen, Germany) and energy-dispersive X-ray spectroscopy (SEM-EDS, Zeiss Gemini 300, Oberkochen, Germany) analysis was performed. The morphology of NiCo_2_S_4_/N-CDs/RGO hydrogel was observed using a transmission electron microscope (FEI Tecnai G2 F20, Hillsboro, OR, USA) at an acceleration voltage of 200 kV. The crystal structures of N-CDS/RGO, NiCo_2_S_4_/RGO and NiCo_2_S_4_/N-CDs/RGO hydrogels were analyzed using An Ultima IV X-ray diffrotometer (Ultima IV, Tokyo, Japan) with Cu Kα radiation source at a wavelength of 1.540 Å. The surface chemistry and elemental composition of the samples were analyzed using X-ray photoelectron spectroscopy (Kratos AXIS SUPRA, Manchester, UK) with an Al Kα radiation source.

### 2.6. Electrochemical Measurements

The electrochemical properties of electrode materials and supercapacitors were studied by different electrochemical programs (CV, GCD and EIS) using a CHI660D electrochemical workstation (Shanghai Chenhua, China) in 3 M KOH electrolyte solution. In the three-electrode system, a platinum plate and Hg/HgO electrode were used as counter and reference electrodes, respectively. CV and GCD curves were obtained in the voltage range of 0 to 0.5 V, and EIS curves were obtained in the frequency range of 0.01 Hz to 100 kHz. The mass specific capacitance C_m_ (F g^−1^) was calculated according to the CV and GCD curves, with the calculation formulas as follows:

(1) $$C_{m}=\frac{\int I\left(V\right)dV}{mv∆V}$$

(2) $$C_{m}=\frac{I\cdot ∆t}{m\cdot ∆V}$$

where I (V) is the function of current response with respect to voltage V, ν (mV s^−1^) is the scan rate, ΔV (V) is the voltage window difference, m (g) is the effective mass of the active material, I (A) is the discharge current, and Δt (s) is the discharge time.

The energy density (*E*, Wh kg^−1^) and power density (*P*, W kg^−1^) of the self-assembled supercapacitor are calculated as follows:

(3) $$E=\frac{1}{2}C∆V^{2}$$

(4) $$P=\frac{E}{∆t}$$

Among them, C (F g^−1^) is the mass specific capacitance of the supercapacitor, ΔV (V) is the potential window, and Δt (h) is the discharge time.

## 3. Results and Discussion

**Figure 2:**
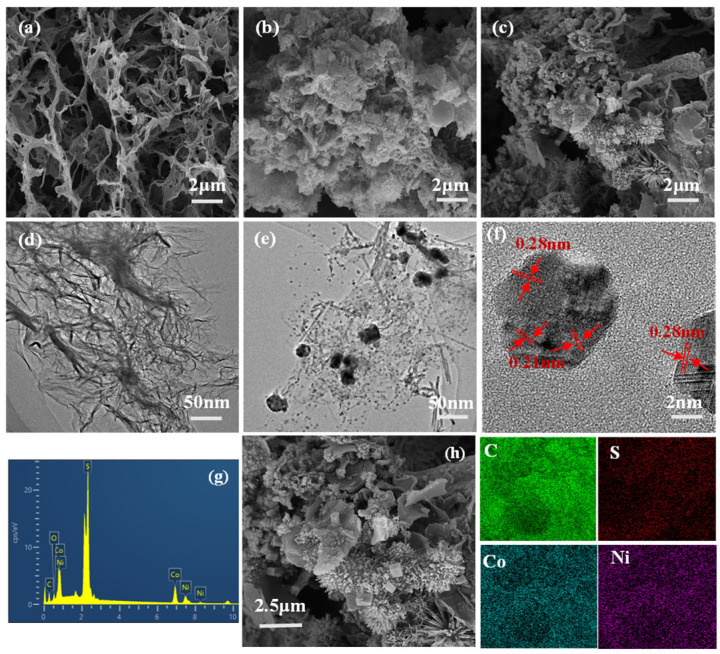
SEM images of (**a**) GH, (**b**) NiCo_2_S_4_/RGO, (**c**) NiCo_2_S_4_/N-CDs/RGO; TEM images of (**d**) GH; (**e**) NiCo_2_S_4_/N-CDs/RGO; (**f**) HRTEM image of NiCo_2_S_4_/N-CDs/RGO; (**g**,**h**) EDS element analysis and mapping images of NiCo_2_S_4_/N-CDs/RGO hydrogel.

The morphology and microstructure of the samples were studied by SEM and TEM. Figure 2a–c show the SEM images of GH, NiCo_2_S_4_/RGO, and NiCo_2_S_4_/N-CDS/RGO hydrogels, respectively. In GH hydrogels (Figure 2a), graphene sheets are interlaced with each other and have an abundant pore structure. It can be clearly seen from Figure 2b that a large number of NiCo_2_S_4_ particles are clustered on the graphene sheets, and the graphene sheets provide sufficient space for the synthesis of NiCo_2_S_4_. The addition of N-CDs increases the interlayer spacing of graphene sheets. Meanwhile, the abundant functional groups on the surface of N-CDs interact with Ni (II) and Co (III) cations, which is beneficial to the synthesis of NiCo_2_S_4_ ions (Figure 2c). During the hydrothermal process, the oxygen-containing functional groups on graphene oxide are reduced, the sp^2^ structure is continuously restored, and the van der Waals force between graphene sheets is also continuously enhanced, which promotes the self-assembly of graphene to form a hydrogel. At the same time, NiCo_2_S_4_ particles grow in situ between graphene sheets and are sealed in graphene sheets with N-CDs to form NiCo_2_S_4_/N-CDS/RGO hybrid. From the TEM images of GH and NiCo_2_S_4_/N-CDS/RGO hydrogels (Figure 2d,e), it can be seen that the size of spherical N-CDs is about 2~3 nm, and the size of NiCo_2_S_4_ nanoparticles is in the range of 20~40 nm; they are uniformly fixed in the graphene sheets, which is beneficial to reduce the stacking of graphene sheets. The HRTEM image of NiCo_2_S_4_/N-CDS/RGO (Figure 2f) shows lattice fringes, with interlayer distances of 0.28 nm and 0.21 nm corresponding to crystal planes of cubic NiCo_2_S_4_ (511) and graphite (100), respectively. In addition, Figure 2g,h show EDS and the corresponding element mapping of the NiCo_2_S_4_/N-CDS/RGO composite, confirming the existence of Ni, Co and S elements. According to EDS, the atomic mass ratio of Ni/Co/S is about 1:2:4, also indicating the formation of NiCo_2_S_4_.

**Figure 3:**
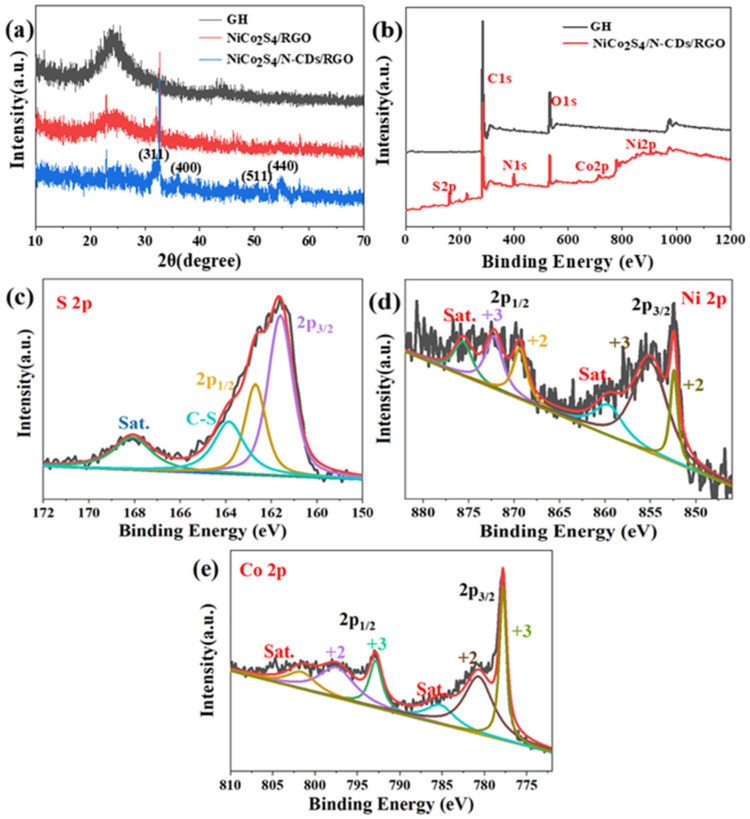
(**a**) XRD patterns of GH, NiCo_2_S_4_/RGO and NiCo_2_S_4_/N-CDs/RGO; (**b**) XPS spectra survey of GH and NiCo_2_S_4_/N-CDs/RGO; (**c**) S 2p spectrum, (**d**) Ni 2p spectrum and (**e**) Co 2p spectrum of NiCo_2_S_4_/N-CDs/RGO.

The crystalline properties of the GH, NiCo_2_S_4_/RGO and NiCo_2_S_4_/N-CDs/RGO composites were analyzed and compared by XRD. As shown in Figure 3a, a wide peak was detected at 2θ = 26° for the GH sample, corresponding to the (002) crystallographic plane of the graphitic structure, indicating the successful reduction in graphene oxide during the hydrothermal reaction. The appearance of (311), (400), (440), and (511) diffraction peaks in NiCo_2_S_4_/RGO and NiCo_2_S_4_/N-CDs/RGO proves the successful synthesis of NiCo_2_S_4_, matching JCPDS20-0782 [2]. Notably, the diffraction peak intensity of NiCo_2_S_4_/N-CDs/RGO prepared by adding carbon dots to the NiCo_2_S_4_ and RGO systems is significantly enhanced, suggesting that carbon dots benefit NiCo_2_S_4_ nucleation and growth. The chemical bond state of each element in the samples was further analyzed by XPS. Figure 3b shows the XPS spectra of GH and NiCo_2_S_4_/N-CDs/RGO. Compared to GH, new characteristic peaks appear in NiCo_2_S_4_/N-CDs/RGO, corresponding to N 1s, Ni 2p, Co 2p and S 2p, confirming the successful doping of N, Ni, Co, and S into NiCo_2_S_4_/N-CDs/RGO. The XPS spectra of Ni, Co and S elements were fitted by Gaussian fitting. Figure 3c shows the S 2p spectrum; peaks at 161.3 eV and 162.7 eV correspond to S 2p_3/2_ and S 2p_1/2_ of the metallic–sulfur bond respectively, while the peak at 163.8 eV relates to heterocyclic configuration (C-S), indicating covalent bonding between graphene and sulfur with successful sulfur doping into the carbon-based material [30]. Figure 3d shows the refined Ni 2p spectrum after Gaussian fitting. Characteristic peaks at 870.1 eV and 852.4 eV located at 2p_1/2_ and 2p_3/2_ belong to Ni^2+^, while two characteristic peaks at 872.2 eV and 855.0 eV relate to Ni^3+^. Similarly, the fine spectrum of Co 2p is shown in Figure 3e, characteristic peaks of binding energy at 780.7 eV and 797.4 eV indicate the presence of Co^2+^ in the sample, while characteristic peaks at 777.8 eV and 792.8 eV can be attributed to the Co^3+^ valence state.

Cyclic voltammetry (CV) tests were performed on GH, NiCo_2_S_4_/RGO and NiCo_2_S_4_/N-CDs/RGO composites in a 3 M KOH electrolyte solution, using a platinum counter electrode and Ag/AgCl reference electrode. The oxidation–reduction reaction of NiCo_2_S_4_ in alkaline electrolyte generates NiSOH, CoSOH, and CoSO. The reaction process can be described by the following equation:

NiCo_2_S_4_+2OH^−^ ⇋ NiS_4-2x_OH+2CoS_x_OH+e^−^; CoS_x_OH+OH^−^ ⇋ CoS_x_O+H_2_O+e^−^

**Figure 4:**
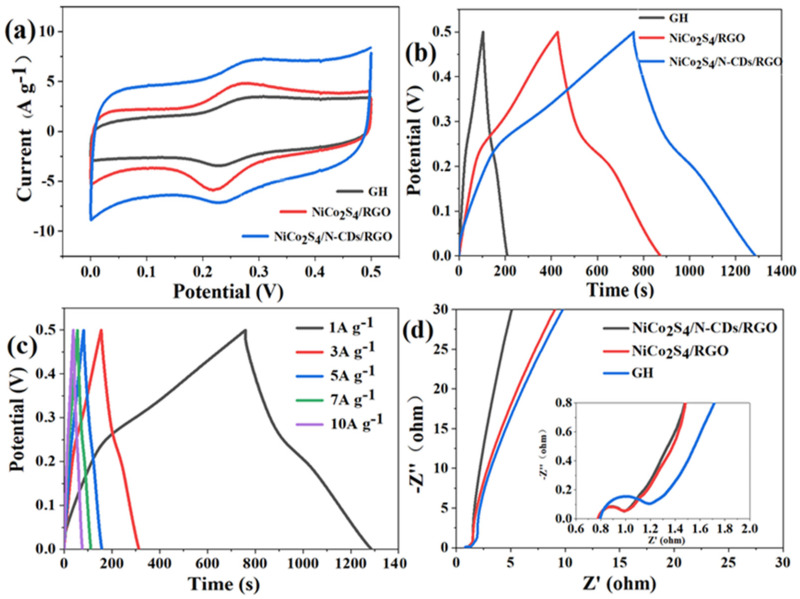
(**a**) CV curves of GH, NiCo_2_S_4_/RGO and NiCo_2_S_4_/N−CDs/RGO with scan rate of 5 mV s^−1^; (**b**) GCD curves of GH, NiCo_2_S_4_/RGO and NiCo_2_S_4_/N-CDs/RGO at 1 A g^−1^; (**c**) GCD curves of NiCo_2_S_4_/N−CDs/RGO at different current densities of 1~10 A g^−1^; (**d**) Nyquist plots of GH, NiCo_2_S_4_/RGO and NiCo_2_S_4_/N−CDs/RGO electrodes in 3 M KOH solution (Inset: the image of the high frequency region).

During charging, ions in the electrolyte diffuse into the NiCo_2_S_4_ lattice and undergo conversion, generating pseudocapacitance. Excess ions form an electrochemical double-layer capacitance on the carbon material surface. As shown in Figure 4a, the CV curves of NiCo_2_S_4_/RGO and NiCo_2_S_4_/N-CDs/RGO exhibit a pair of redox peaks on a rectangular shape, mainly due to the reversible redox reactions of Ni^2+^/Ni^3+^ and Co^2+^/Co^3+^ with OH^−^ anions, increasing pseudocapacitance [22,44]. Compared to GH and NiCo_2_S_4_/RGO, the CV curve of NiCo_2_S_4_/N-CDs/RGO encloses a larger area, indicating higher specific capacitance. This can be attributed to the edge effect of nitrogen-doped carbon dots, enhancing ion diffusion. Figure 4b is the galvanostatic charge–discharge (GCD) diagram in the same potential range as the CV curve. Per Formula (2), the specific capacitances of GH, NiCo_2_S_4_/RGO, and NiCo_2_S_4_/N-CDs/RGO are 235 F g^−1^, 877 F g^−1^, and 1050 F g^−1^, respectively. At current densities from 1 to 10 A g^−1^, the GCD curves of NiCo_2_S_4_/N-CDs/RGO (Figure 4c) show a specific capacitance of 708 F g^−1^ even at 10 A g^−1^ with a retention rate of 67%. Figure 4d shows the Nyquist plot tested from 0.1 Hz to 100 kHz, with an inset expanded view of the high frequency region. In the low-frequency region, the approximately vertical lines indicate near-ideal electric double-layer capacitance behavior [45], explaining the good rate performance of NiCo_2_S_4_/N-CDs/RGO. Compared to GH, NiCo_2_S_4_/RGO, and NiCo_2_S_4_/N-CDs/RGO have smaller arc radius in the high-frequency region, indicating the rapid transport and diffusion of ions/electrons between the NiCo_2_S_4_ and electrolyte interface and within the composite electrode [46,47].

**Figure 5:**
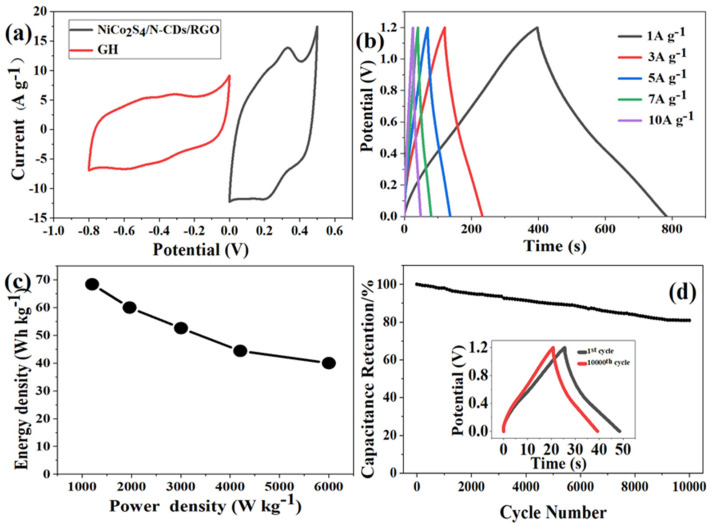
(**a**) CV curves of the NiCo_2_S_4_/N−CDs/RGO and GH electrodes at 25 mV s^−1^; (**b**) GCD curves of NiCo_2_S_4_/N−CDs/RGO//GH supercapacitor at different current densities; (**c**) Ragone plot of the supercapacitor; (**d**) capacitance retention percentage of the supercapacitor for 10,000 charging/discharging cycles (inset: the GCD curves of the 1st and 8000th cycles).

To further evaluate the practical application of NiCo_2_S_4_/N-CDs/RGO electrodes in supercapacitors, NiCo_2_S_4_/N-CDs/RGO and GH hydrogels were used as positive and negative electrodes, respectively, and a novel NiCo_2_S_4_/N-CDs/RGO//GH asymmetric supercapacitor was assembled. To determine the voltage range of the assembled capacitor, CV electrochemical tests were performed on GH and NiCo_2_S_4_/N-CDs/RGO electrodes in a three-electrode system at a scanning rate of 25 mV s^−1^. As shown in Figure 5a, the NiCo_2_S_4_/N-CDs/RGO electrode exhibits redox behavior, and GH exhibits typical dual capacitance properties. The voltage windows of NiCo_2_S_4_/N-CDs/RGO and GH are −0.8~0 V and 0~0.5 V, respectively. We can infer that the flexible energy storage device can provide a stable voltage of 1.3 V. In Figure 5b, the potential–time curves are nearly symmetrical at all current densities, indicating a high Coulombic efficiency of assembled device. The energy and power densities of NiCo_2_S_4_/N-CDs/RGO//GH were calculated according to Formulas (3) and (4). As shown in Figure 5c, the energy density of the device reaches 68.4 Wh kg^−1^ at 1201 W kg^−1^, and 40 Wh kg^−1^ at 6000 W kg^−1^. Figure 5d shows NiCo_2_S_4_/N-CDs/RGO//GH retains 82% of its capacitance after 10,000 constant current charge–discharge cycles at a current density of 10 A g^−1^. After 10,000 cycles, the charge–discharge time of GCD curve is reduced, which may be due to a loss of adhesion between some active substances and gel electrolyte during the charge–discharge cycle (as shown in the illustration).

## 4. Conclusions

In summary, three different hydrogel electrode materials were prepared by a one-step hydrothermal method: GH, NiCo_2_S_4_/RGO, and NiCo_2_S_4_/N-CDs/RGO. It was found that NiCo_2_S_4_/N-CDs/RGO exhibits excellent electrochemical performance, with a specific capacitance of 1050 F g^−1^ at a current density of 1 A g^−1^. This is mainly attributed to the fact that N-CDs not only enhanced the bonding strength of NiCo_2_S_4_ and graphene, but also contributed to the improvement of the specific capacitance of the composite due to its own electrical conductivity. In addition, a solid-state asymmetric supercapacitor assembled with NiCo_2_S_4_/N-CDs/RGO as the positive electrode exhibited excellent energy density (68.4 Wh kg^−1^) and cycle stability (its capacitance remains 82% after 10,000 constant current charge–discharge cycles).

## Data Availability

Data are contained within the article.

## References

[1] Weng Z., Su Y., Wang D.-W., Li F., Du J., Cheng H.-M. (2011). Graphene-Cellulose Paper Flexible Supercapacitors. Adv. Energy Mater..

[2] Kumbhar V.S., Chodankar N.R., Lee K., Kim D.H. (2019). Insights into the interfacial nanostructuring of NiCo_2_S_4_ and their electrochemical activity for ultra-high capacity all-solid-state flexible asymmetric supercapacitors. J. Colloid Interface Sci..

[3] Bi H., He X., Zhang H., Li H., Xiao N., Qiu J. (2021). N, P co-doped hierarchical porous carbon from rapeseed cake with enhanced supercapacitance. Renew. Energy.

[4] Ma J.-S., Yang H., Kubendhiran S., Lin L.-Y. (2022). Novel synthesis of sulfur-doped graphitic carbon nitride and NiCo_2_S_4_ composites as efficient active materials for supercapacitors. J. Alloys Compd..

[5] Yuan T., Zhang Z., Liu Q., Liu X.-T., Tao S.-Q., Yao C.-l. (2024). Cellulose nanofiber/MXene (Ti_3_C_2_T_x_)/liquid metal film as a highly performance and flexible electrode material for supercapacitors. Int. J. Biol. Macromol..

[6] Vidyadharan B., Aziz R.A., Misnon I.I., Anil Kumar G.M., Ismail J., Yusoff M.M., Jose R. (2014). High energy and power density asymmetric supercapacitors using electrospun cobalt oxide nanowire anode. J. Power Sources.

[7] Saha D., Li Y., Bi Z., Chen J., Keum J.K., Hensley D.K., Grappe H.A., Meyer H.M., Dai S., Paranthaman M.P. (2014). Studies on supercapacitor electrode material from activated lignin-derived mesoporous carbon. Langmuir.

[8] Volkov A.I., Ivanov A.V., Vereshchagin A.A., Novoselova J.V., Tolstopjatova E.G., Kondratiev V.V. (2022). Electrochemical deposition of PEDOT/MoS2 composite films for supercapacitors. Synth. Met..

[9] Zhong W., Su W., Li P., Li K., Wu W., Jiang B. (2024). Preparation and research progress of lignin-based supercapacitor electrode materials. Int. J. Biol. Macromol..

[10] Gao X., Han G., Song H., Chang Y., Xiao Y., Zhang Y., Liu C., Li H. (2019). Purified nitrogen-doped reduced graphene oxide hydrogels for high-performance supercapacitors. J. Electroanal. Chem..

[11] Xing H., He W., Liu Y., Long G., Sun Y., Feng J., Feng W., Zhou Y., Zong Y., Li X. (2021). Ultrathin and Highly Crumpled/Porous CoP Nanosheet Arrays Anchored on Graphene Boosts the Capacitance and Their Synergistic Effect toward High-Performance Battery-Type Hybrid Supercapacitors. ACS Appl. Mater. Interfaces.

[12] Nithya V.D. (2021). A review on holey graphene electrode for supercapacitor. J. Energy Storage.

[13] Chen X., Ma J., Sun X., Zhao C., Li J., Li H. (2024). SiC and N, S-doped carbon nanosheets and lignin-enhanced organohydrogel for low-temperature tolerant solid-state supercapacitors. Int. J. Biol. Macromol..

[14] Kumar R., Thangappan R. (2022). Electrode material based on reduced graphene oxide (rGO)/transition metal oxide composites for supercapacitor applications: A review. Emergent Mater..

[15] Frackowiak E., Foroutan Koudahi M., Tobis M. (2021). Electrochemical Capacitor Performance of Nanotextured Carbon/Transition Metal Dichalcogenides Composites. Small.

[16] Pramanik A., Maiti S., Sreemany M., Mahanty S. (2016). Carbon Doped MnCo_2_S_4_ Microcubes Grown on Ni foam as High Energy Density Faradaic Electrode. Electrochim. Acta.

[17] Cao X., He J., Li H., Kang L., He X., Sun J., Jiang R., Xu H., Lei Z., Liu Z.H. (2018). CoNi_2_S_4_ Nanoparticle/Carbon Nanotube Sponge Cathode with Ultrahigh Capacitance for Highly Compressible Asymmetric Supercapacitor. Small.

[18] Wu J., Dou S., Shen A., Wang X., Ma Z., Ouyang C., Wang S. (2014). One-step hydrothermal synthesis of NiCo_2_S_4_–rGO as an efficient electrocatalyst for the oxygen reduction reaction. J. Mater. Chem. A.

[19] Gayathri V., John Peter I., Ramachandran K., Karazhanov S., Raja Mohan C. (2021). Graphene Quantum Dots Embedded in NiCo_2_S_4_/MWCNT Nanocomposite as a Promising Candidate for Supercapacitors and *I*_3_^−^ Reduction in Dye-Sensitized Solar Cells. Energy Fuels.

[20] Liu J., Chen X., Zhu Y., Chen R., Yuan W. (2021). NiCo_2_S_4_/nitrogen and sulfur dual-doped three-dimensional holey-reduced graphene oxide composite architectures as high-rate battery-type cathode materials for hybrid supercapacitors. Vacuum.

[21] Saravanakumar B., Jayaseelan S.S., Seo M.K., Kim H.Y., Kim B.S. (2017). NiCo_2_S_4_ nanosheet-decorated 3D, porous Ni film@Ni wire electrode materials for all solid-state asymmetric supercapacitor applications. Nanoscale.

[22] Huang Y., Shi T., Zhong Y., Cheng S., Jiang S., Chen C., Liao G., Tang Z. (2018). Graphene-quantum-dots induced NiCo_2_S_4_ with hierarchical-like hollow nanostructure for supercapacitors with enhanced electrochemical performance. Electrochim. Acta.

[23] Yin J., Wang Y., Meng W., Zhou T., Li B., Wei T., Sun Y. (2017). Honeycomb-like NiCo_2_S_4_ nanosheets prepared by rapid electrodeposition as a counter electrode for dye-sensitized solar cells. Nanotechnology.

[24] Han H., Song Y., Zhang Y., Kalimuldina G., Bakenov Z. (2021). NiCo_2_S_4_ Nanocrystals on Nitrogen-Doped Carbon Nanotubes as High-Performance Anode for Lithium-Ion Batteries. Nanoscale Res. Lett..

[25] Torrisi L., Cutroneo M., Havranek V., Silipigni L., Fazio B., Fazio M., Di Marco G., Stassi A., Torrisi A. (2019). Self-supporting graphene oxide films preparation and characterization methods. Vacuum.

[26] Sonia F.J., Kalita H., Aslam M., Mukhopadhyay A. (2017). Correlations between preparation methods, structural features and electrochemical Li-storage behavior of reduced graphene oxide. Nanoscale.

[27] Zhu G., He Z., Chen J., Zhao J., Feng X., Ma Y., Fan Q., Wang L., Huang W. (2014). Highly conductive three-dimensional MnO_2_-carbon nanotube-graphene-Ni hybrid foam as a binder-free supercapacitor electrode. Nanoscale.

[28] Liu R., Wan L., Liu S., Pan L., Wu D., Zhao D. (2015). An Interface-Induced Co-Assembly Approach Towards Ordered Mesoporous Carbon/Graphene Aerogel for High-Performance Supercapacitors. Adv. Funct. Mater..

[29] Liu X., Zou S., Liu K., Lv C., Wu Z., Yin Y., Liang T., Xie Z. (2018). Highly compressible three-dimensional graphene hydrogel for foldable all-solid-state supercapacitor. J. Power Sources.

[30] Li B., Tian Z., Li H., Yang Z., Wang Y., Wang X. (2019). Self-supporting graphene aerogel electrode intensified by NiCo_2_S_4_ nanoparticles for asymmetric supercapacitor. Electrochim. Acta.

[31] Dai Y., Liu Z., Bai Y., Chen Z., Qin J., Feng F. (2018). A novel highly fluorescent S, N, O co-doped carbon dots for biosensing and bioimaging of copper ions in live cells. RSC Adv..

[32] Rai S., Singh B.K., Bhartiya P., Singh A., Kumar H., Dutta P.K., Mehrotra G.K. (2017). Lignin derived reduced fluorescence carbon dots with theranostic approaches: Nano-drug-carrier and bioimaging. J. Lumin..

[33] Chen W., Hu C., Yang Y., Cui J., Liu Y. (2016). Rapid Synthesis of Carbon Dots by Hydrothermal Treatment of Lignin. Matererials.

[34] Xiong J., Pan Q., Zheng F., Xiong X., Yang C., Hu D., Huang C. (2018). N/S Co-doped Carbon Derived From Cotton as High Performance Anode Materials for Lithium Ion Batteries. Front. Chem..

[35] Lu Z., Zhang Y., Sun M., Zou P., Wang X., Wang Y., Huang Q., Chen H., Ye J., Rao H. (2021). N-doped carbon dots regulate porous hollow nickel-cobalt sulfide: High-performance electrode materials in supercapacitor and enzymeless glucose sensor. J. Power Sources.

[36] Qu F., Pei H., Kong R., Zhu S., Xia L. (2017). Novel turn-on fluorescent detection of alkaline phosphatase based on green synthesized carbon dots and MnO_2_ nanosheets. Talanta.

[37] Liu Y., Duan W., Song W., Liu J., Ren C., Wu J., Liu D., Chen H. (2017). Red Emission B, N, S-co-Doped Carbon Dots for Colorimetric and Fluorescent Dual Mode Detection of Fe^3+^ Ions in Complex Biological Fluids and Living Cells. ACS Appl. Mater. Interfaces.

[38] Sun L., Mo Z., Li Q., Zheng D., Qiu X., Pan X. (2021). Facile synthesis and performance of pH/temperature dual-response hydrogel containing lignin-based carbon dots. Int. J. Biol. Macromol..

[39] Uddin M.-J., Alaboina P.K., Zhang L., Cho S.-J. (2017). A low-cost, environment-friendly lignin-polyvinyl alcohol nanofiber separator using a water-based method for safer and faster lithium-ion batteries. Mater. Sci. Eng. B.

[40] Moreno Araújo Pinheiro Lima R., de Oliveira H.P. (2020). Carbon dots reinforced polypyrrole/ graphene nanoplatelets on flexible eggshell membranes as electrodes of all-solid flexible supercapacitors. J. Energy Storage.

[41] Zhang W., Yin J., Lin Z., Lin H., Lu H., Wang Y., Huang W. (2015). Facile preparation of 3D hierarchical porous carbon from lignin for the anode material in lithium ion battery with high rate performance. Electrochim. Acta.

[42] Svinterikos E., Zuburtikudis I., Al-Marzouqi M. (2020). Electrospun Lignin-Derived Carbon Micro- and Nanofibers: A Review on Precursors, Properties, and Applications. ACS Sustain. Chem. Eng..

[43] Cao N., Zhang Y. (2015). Study of Reduced Graphene Oxide Preparation by Hummers’ Method and Related Characterization. J. Nanomater..

[44] Farshadnia M., Ensafi A.A., Zarean Mousaabadi K., Rezaei B. (2022). Design and synthesis of three-dimensional CoNi_2_S_4_@MoS_2_@rGO nanocomposites and its application in electrochemical supercapacitors. J. Alloys Compd..

[45] Kongthong T., Poochai C., Sriprachuabwong C., Tuantranont A., Nanan S., Meethong N., Pakawatpanurut P., Amornsakchai T., Sodtipinta J. (2022). Microwave-assisted synthesis of nitrogen-doped pineapple leaf fiber-derived activated carbon with manganese dioxide nanofibers for high-performance coin- and pouch-cell supercapacitors. J. Sci. Adv. Mater. Devices.

[46] Feng L., Chang Y., Song H., Hou W., Li Y., Zhao Y., Xiao Y., Han G. (2022). N, S co-doped porous carbon with high capacitive performance derived from heteroatom doped phenolic resin. J. Electroanal. Chem..

[47] Liu C., Hou Y., Li Y., Xiao H. (2022). Heteroatom-doped porous carbon microspheres derived from ionic liquid-lignin solution for high performance supercapacitors. J. Colloid Interface Sci..

